# The effect of hydroalcoholic extract of *Nigella Sativa* seed on dehydroepiandrosterone-induced polycystic ovarian syndrome in rats: An experimental study

**DOI:** 10.18502/ijrm.v19i3.8575

**Published:** 2021-03-21

**Authors:** Samira Khani, Maasoume Abdollahi, Azam Khalaj, Hamid Heidari, Somaye Zohali

**Affiliations:** ^1^Neuroscience Research Center, Qom University of Medical Sciences, Qom, Iran.; ^2^Department of Anatomical Sciences, Medical Sciences Faculty, Tarbiat Modares University, Tehran, Iran.; ^3^Department of Physiology, School of Medicine, Qom University of Medical Sciences, Qom, Iran.; ^4^Cellular and Molecular Research Center, Qom University of Medical Sciences, Qom, Iran.; ^5^Department of Physiology and Pharmacology, Faculty of Medicine, Qom University of Medical Sciences, Qom, Iran.; ^6^Student Research Committee, Qom University of Medical Sciences, Qom, Iran.

**Keywords:** Nigella sativa seed, Oxidative stress, Insulin resistance, Polycystic ovary syndrome, Rat.

## Abstract

**Background:**

Polycystic ovary syndrome (PCOS) is one of the most common endocrine disorders among women.

**Objective:**

The aim of this study was to investigate the therapeutic effect of hydroalcoholic extract of *Nigella sativa* (*N. sativa*) seed as a plant, the consumption of which has been recommended in Islam, on dehydroepiandrosterone (DHEA)-induced PCOS rats.

**Materials and Methods:**

This experimental study was carried out on 36 Wistar female rats (3 wk, 60 ± 10 gr). Then rats were divided into 6 groups (n = 6/each): control; PCOS-induced (DHEA 60 mg/kg/sc); PCOS+ Metformine (30 mg/kg); and three experimental groups receiving DHEA + hydroalcoholic extract of *N. sativa* seeds in doses of 50, 100 and 200 mg/kg, respectively. Blood samples were taken for the evaluation of sexual hormones, oxidative stress, glucose, and insulin after 30 days of treatment. Ovarian tissue was used for histopathological study.

**Results:**

The serum levels of luteinizing hormone, testosterone, glucose, insulin resistance, malondialdehyde, and insulin (p ≤ 0.001) and estrogen increased while the levels of progesterone (p = 0.01) and antioxidant enzymes in the PCOS group decreased (p ≤ 0.001).

**Conclusion:**

The administration of the *N. sativa* extract to the PCOS rats resulted in remarkable changes in the serumic factors relative to the PCOS group. In addition, the extract improved the structure of the ovarian tissue in the PCOS rat. The histopathological results which are in accordance with biochemical findings imply that *N. sativa* seed could be useful in the treatment of PCOS, the higher doses of the extract being more effective.

## 1. Introduction

Polycystic ovary syndrome (PCOS) is one of the most common endocrine disorders among women (1). The causes of PCOS can be attributed to defects in the hypothalamic-pituitary function and insulin action (2).

Increased androgens and obesity, insulin resistance, and type 2 diabetes, and oxidative stress are the long-term consequences of this syndrome (3, 4). Disturbance in antioxidant systems may lead to pathological outcomes such as PCOS and disruption of the synthesis of ovarian steroids in women (5). An impaired release of gonadotropin associated with enhanced secretion of luteinizing hormone (LH) compared to the follicle-stimulating hormone (FSH) are observed in these patients (6). Several medical treatments are available for PCOS, however, most of them are temporary and not definitive treatment. Given the side effects of these drugs, identifying and providing alternative drugs are very important (7).

In this study, the effects of *Nigella sativa* seeds (*N. sativa *also known as black cumin) used in traditional medicine have been investigated. The seeds of this plant have antimicrobial and antifungal properties, in addition to being menstrual regulators and milk boosters (8), and are used in Iranian traditional medicine for treating several inflammatory and painful disorders (9).

The compounds isolated from *N. sativa,* including thymoquinone, t-anethole, carvacrol, and 4-terpinol, have detectable free radical scavenging, antioxidant, and anti-inflammatory properties (10). Regarding the effect of this plant on the hypothalamic-pituitary axis and to determine its effect on PCOS for the first time, we decided to evaluate the effect of the extract of this seed on the histological and hormonal levels and the appropriate dosage of the extract to reduce ovarian cysts, insulin resistance, and oxidative stress in rats model of the PCOS.

## 2. Materials and Methods 

### Animals

In this experimental study, which was carried out in 2017 at the Qom University of Medical Sciences, 36 female Wistar rats (60 ± 10 gr, aged 21 days) (11) obtained from the animal house of the Qom Medical University were used. The rats were housed in a room with controlled temperature (23 ± 2°C) and luminosity (12-hr light/dark cycles) with free access to food and water.

### Experimental groups

Rats were randomly divided into six groups (n = 6/each):

Group 1 (control): rats received no medication;

Group 2 (PCOS): dehydroepiandrosterone (DHEA)-induced PCOS-control or PCOS;

Group 3 (PCOS+ Met) as a positive control: rats with PCOS receiving 30 mg/100 g of Metformin (Chemidarou Co. Tehran, Iran) through gavage for one month;

Groups 4 (PCOS + N.S 50), Groups 5 (PCOS + N.S 100) and Groups 6 (PCOS + N.S 200): PCOS rats which received a hydroalcoholic extract of *N. Sativa* seeds in doses of 50, 100, and 200 mg/kg respectively, through gavage for one month.

### Plant preparation and extract

The seeds of *N. sativa *were purchased from a local market in Qom (Iran) and authenticated scientifically by the Department of Medicinal Plants, Qom University of Medical Science (voucher specimen: PMP-735). The maceration method was used for the preparation of the extract. To prepare the hydroalcoholic extract, 50 gr of seed powder was soaked in 200 ml of hydroalcoholic solvent (distilled water-ethanol 96% mixture; 60-40) and kept for 72 hr in a closed container at room temperature. Then, the contents of the container were filtered using a Buchner funnel (Whatman's filter paper, No. 1), after removing the solvent and drying at room temperature, to obtain the semisolid mass which was kept at 4°C prior to use (12, 13).

### Induction of PCOS model

PCOS was induced by a subcutaneous injection of DHEA (60 mg/kg, Cayman, USA) dissolved in sesame oil for 3 wk. According to other similar studies on PCOS induction, after 21 days of DHEA injection, the rats were subjected to vaginal smear tests to determine the irregularity of the estrous cycle and the occurrence of persistent vaginal carnification (PVC), which is one of the symptoms of follicular cysts in the ovary (14). Moreover, the induction of PCOS was confirmed by serological tests (such as increase in LH, testosterone, and HOMA-IR levels and decrease in FSH and progesterone levels) and histomorphometric studies (wherein one or both ovaries can contain multiple small, immature ovarian follicles as cysts).

### Serum analysis

At the end of the treatment period and 24 hr after the last gavage, the animals were subjected to deep anesthesia with diethyl ether. Blood samples were directly collected by cardiac puncture and centrifuged (Eppendorf centrifuge 5702, Germany) at 3,000 rpm for 15 min to separate serum samples (Eppendorf centrifuge 5702, Germany) (15, 16). Serum levels of FSH, LH, progesterone, estradiol, and testosterone were measured by the ELISA kit (Pars Azmoon, Iran) according to the manufacturer's kit.

Moreover, while the fasting blood glucose (FBG) was determined through a lateral tail vein using an Elegance glucometer (CT-X10, Convergent Technologies, Germany) and serum insulin levels were also measured using commercial ELISA kit (Monobimd, California, USA).

Furthermore, the indices of insulin resistance at the beginning and end of the treatment using HOMA-IR (Homeostasis Model Assessment of Insulin Resistance) were calculated according to the following equation: fasting glucose (mg/dl) × fasting insulin (µIU/ml)/405 (17).

### Antioxidant assessment

Serum antioxidant enzyme superoxide dismutase (SOD) (ZB-SOD-96A), glutathione peroxidase (GPX) (ZB-GPX-96A) (18), and catalase (CAT) (ZB-CAT-96A) activities were measured using ELISA assays kits (ZellBio GmbH, Germany), according to the provider's instructions.

Also, malondialdehyde (MDA) level as a biomarker of lipid peroxidation was assayed according of the kit protocol (ZB-MDA-96A).

### Ovarian morphology

After the blood collection, uterus and ovaries were removed from the body of the rats for histopathological study.

After removing the adherent connective adipose tissue, then the ovaries tissues were embedded in a solution containing 10% formaldehyde for at least 48 hr for fixation. Afterwards, the tissue preparation phase were carried out according to the standard protocols. The samples were dehydrated, embedded in paraffin, serially sectioned, and stained with hematoxylin and eosin. Follicles were counted in all section and classified according to the study of Luo and collaborators (19).

### Ethical considerations

Procedures related to the animals were approved by the Ethical Committee of Qom University of Medical Sciences (code: IR.MUQ.REC.1394.119) and the Ethical Principles of work on animals were taken in accordance with the agenda of the Ethics Committee of the Medical Sciences University.

### Statistical analysis

Data were indicated as mean ± SEM. Statistical evaluation was done by analysis of variance (one-way ANOVA), followed by Tukey's test. The test was performed using the SPSS Statistics, v. 17.01 (Statistical Package for the Social Sciences, SPSS Inc., Chicago, USA). P < 0.05 was considered statistically significant.

## 3. Results 

### Effect of hydroalcoholic extract of *N. sativa* seed on hormonal levels

#### Serum LH and FSH levels 

The results of this study showed that LH level increased in PCOS group (p < 0.001). The treatment of PCO animals with metformin and doses of 50, 100, and 200 mg/kg of the *N. sativa* extract decreased LH levels (p < 0.001 and p = 0.05 respectively). The level of FSH decreased in PCOS rats, while it and FSH increased in other groups (Table I).

#### Serum testosterone level

The testosterone levels elevated in PCOS rats (p < 0.001) and treatment with metformin and a dose of 200 mg/kg *N. sativa* reduced it (p = 0.003 and p = 0.01, respectively) (Table I).

#### Serum progesterone and estrogen levels

The mean serum level of progesterone downregulated in the PCOS group (p = 0.01). Receiving a dose of 200 mg/kg of *N. sativa* extract increased the progesterone level (p = 0.03). Data of the present study showed that the level of estrogen upregulated in the PCOS group while it decreased in all treatment groups (Table I).

#### Glucose, insulin levels, and HOMA-IR 

Following the induction of PCOS, the insulin and FBG levels increased (p = 0.001). The treatment of PCO rats with metformin and a dose of 200 mg/kg of *N. sativa* extract decreased the FBG levels (p = 0.03), while the doses of 100 and 200 mg/kg (p = 0.03 and p = 0.002, respectively) decreased the insulin level. In addition, the treatment of PCO rats with metformin and doses of 100 and 200 mg /kg (p = 0.01, p = 0.02 and, p < 0.001, respectively) of the extract reduced the insulin resistance (Table II).

### Effect of hydroalcoholic extract of *N. sativa* seed on oxidative stress markers

The induction of PCOS decreased the levels of SOD, GPX and CAT (p < 0.001) and increased the level of MDA (p < 0.001) in rat's serum. Treating the PCOS rats with a dose of 200 mg/kg of the *N. sativa* extract increased the SOD (p = 0.003), GPX (p = 0.002), and CAT (p = 0.02) levels. Also, a dose of 100 mg/kg of the extract increased the GPX level (p = 0.02). In addition, receiving the extract in doses 100 (p = 0.006) and 200 mg/kg (p < 0.001) in the PCOS groups decreased MDA levels. Moreover, the administration of metformin in the PCOS group increased the level of SOD (p = 0.01) and CAT (p = 0.12) (Table III).

### Effect of hydroalcoholic extract of *N. sativa* seed on body weight

**Figure 1:**
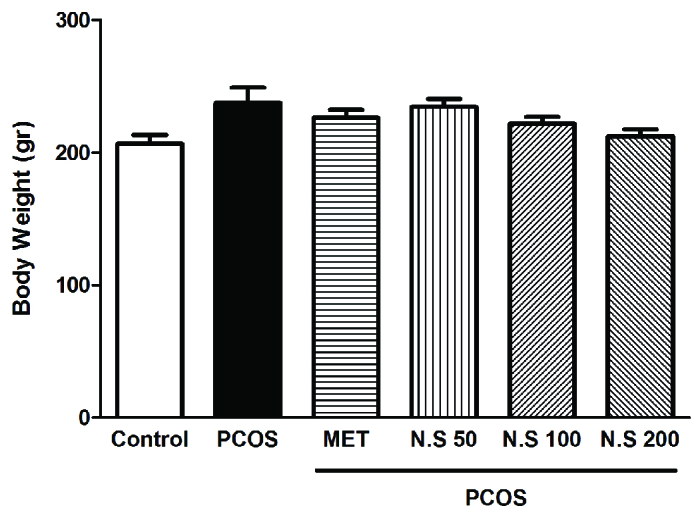
Effect of hydroalcoholic extract of *N. sativa* seed extract on body weight after four weeks in the dehydroepiandrosterone-induced PCOS rats. Results are expressed as Mean ± SEM. CON: Control, PCOS: Polycystic ovarian syndrome, MET: Metformin, N.S: *N. sativa* seed extract.

After 4 wk, the final body weight of PCOS rats increased in the PCOS group compared to the intact control group. Despite the decreased body weight in other groups, there were no significant differences in this item (Figure 1).

### Results of histopathological study 

**Figure 2:**
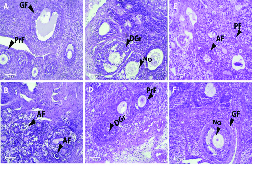
Light microscopy of ovarian tissue samples stained using hematoxylin and eosin. The morphology of follicles in the control (A), PCOS-induced rats (B), 300 mg/kg metformin-treated (C), 50 mg/kg black seed *(N. sativa)-*treated (D), 100 mg/kg black seed *(N. sativa)*-treated (E), and 200 mg/kg black seed *(N. sativa)-*treated (F) groups are shown. Cells were stained with hematoxylin and eosin. PF: Primordial follicle, PRF: Primary follicle, GF: Graafian follicle, AF: Atretic follicle, NO: Normal oocyte, DGr: Degenerated granulosa cells (H&E, 10x).

The normal histological view of the ovary was observed in the control group (Figure 2). The histopathological evaluation of ovarian tissues showed damaging changes in PCOS rats. A slight decrease was observed in the mean number of primordial and primary follicles of the PCOS group in comparison to the control group. The mean number of secondary and graffian follicles were decreased in the PCOS rats (p ≤ 0.001). However, a clear increase in cystic (p ≤ 0.001) and atretic follicles and a decrease in corpus luteum (p ≤ 0.001) was perceived in the PCOS-induced rats. Treatment with metformin and *N. sativa* seed extract, to some extent, improved the pathological changes. A dose of 200 mg/kg of the extract reversed these changes related to primary follicles (p ≤ 0.001). While administration 200 mg/kg of the extract increased the number of graafian follicles (p ≤ 0.001) it decreased the number of cystic follicles (p = 0.01). Receiving dose of 100 mg/kg of extract decreased the atretic follicles (p ≤ 0.001). Moreover administration of doses of 50, 200 mg/kg of extract and also receiving metformin reduced the atretic follicles in comparison to PCO group (p = 0.03, p = 0.04 and p = 0.02, respectively). Also, the doses of 50 and 100 mg/kg of the extract increased the corpus luteum (p ≤ 0.001 and p ≤ 0.001, respectively). Table IV presents the number of different ovarian follicles.

**Table 1:** Effect of hydroalcoholic extract of *N. sativa* seed on FSH, LH, testosterone, progesterone and estradiol levels in dehydroepiandrosterone-induced PCOS rats


[dir=NW,height=0.35in,width=1.16in,innerleftsep=6pt,leftsep=0pt]**Groups** **Factors**	**FSH (mIU/mL)**	**LH (mIU/mL)**	**Estradiol (pg/mL)**	**Testosterone (ng/ml)**	**Progesterone (ng/ml)**
**Control**	15.62 ± 0.68	18.28 ± 0.31***	20.02 ± 2.84	41.01 ± 4.11***	38.40 ± 3.82*
**PCOS**	13.45 ± 0.41	27.76 ± 0.83${}^{\#\#\#}$	32.96 ± 3.72	87.22 ± 6.61${}^{\#\#\#}$	20.16 ± 2.08${}^{\#}$
**PCOS + Met**	14.49 ± 0.58	23 ± 0.94${}^{\#}$**	26.36 ± 3.45	55.92 ± 4.26	32.34 ± 4.31
**PCOS + NS 50**	13.70 ± 0.41	24 ± 1.3${}^{\#}$*	29.24 ± 2.66	76.40 ± 4.86${}^{\#\#}$	25.92 ± 2.45
**PCOS + NS 100**	14.01 ± 0.45	19.80 ± 0.94**	32.70 ± 2.59	68.10 ± 5.94${}^{\#}$	29.52 ± 3.61
**PCOS + NS 200**	14.42 ± 0.66	17.30 ± 0.63 **	24.02 ± 2.45	60.17 ± 3.98*	36.54 ± 0.40*
Data are indicated as Mean ± SEM. Statistical evaluation was done by analysis of variance (one-way ANOVA), followed by Tukey's test. A value of ${}^{*}$p < 0.05, ${}^{**}$p < 0.01, and ${}^{***}$p < 0.00 was considered as significant versus the PCOS control group, A value of ${}^{\#}$p < 0.05, ${}^{\#\#}$p < 0.01, and ${}^{\#\#\#}$p < 0.00 was considered as significant versus the intact control group, PCOS: Polycystic ovarian syndrome, Met: Metformin, NS: *Nigella sativa* seed, FSH: Follicle-stimulating hormone, LH: Luteinizing hormone					

**Table 2:** Effect of hydroalcoholic extract of *N. sativa* seed on insulin, glucose, and HOMA-IR levels in dehydroepiandrosterone-induced PCOS rats


[dir=NW,height=0.35in,width=1.775in,innerleftsep=6pt,leftsep=0pt]**Groups** **Factors**	**FBG (mg/dl)**	**Insulin (µIU/ml)**	**HOMA-IR**
**Control**	93 ± 7.04***	2.95 ± 0.34***	0.66 ± 0.07***
**PCOS**	187.6 ± 12.89${}^{\#\#\#}$	5.97 ± 0.51${}^{\#\#\#}$	2.73 ± 0.20${}^{\#\#\#}$
**PCOS + Met**	128.6 ± 14.74*	4.46 ± 0.51	1.53 ± 0.24*
**PCOS + NS 50**	147.6 ± 13.88	4.74 ± 0.52	1.77 ± 0.34${}^{\#}$
**PCOS + NS 100**	160.6 ± 14.03${}^{\#}$	3.91 ± 0.48*	1.58 ± 0.28*
**PCOS + NS 200**	128.2 ± 12.62*	3.05 ± 0.27**	0.98 ± 0.14***
Data are indicated as Mean ± SEM. Statistical evaluation was done by analysis of variance (one-way ANOVA) followed by Tukey's test. A value of ${}^{*}$p < 0.05, ${}^{**}$p < 0.01, and ${}^{***}$p < 0.00 was considered as significant versus the PCOS control group; A value of ${}^{\#}$p < 0.05, ${}^{\#\#}$p < 0.01, and ${}^{\#\#\#}$p < 0.00 was considered as significant versus the intact control group, PCOS: Polycystic ovarian syndrome, Met: Metformin, NS: *Nigella sativa* seed, FBG: Fasting blood glucose: HOMA-IR: Homeostatic model assessment of insulin resistance			

**Table 3:** Effect of hydroalcoholic extract of *N. sativa* seed on malondialdehyde and antioxidant levels in dehydroepiandrosterone-induced PCOS rats


[dir=NW,height=0.35in,width=1.16in,innerleftsep=6pt,leftsep=0pt]**Groups** **Factors**	**SOD activity (U/mg protein)**	**GPX (U/mg protein)**	**CAT activity (U/mg protein)**	**MDA (nmol/mg protein)**
**Control**	76 ± 6.72***	102.60 ± 7.15***	127.80 ± 9.31***	34.20 ± 3.05***
**PCOS**	33.40 ± 3.74${}^{\#\#\#}$	42.60 ± 4.23${}^{\#\#\#}$	73 ± 5.76${}^{\#\#\#}$	76 ± 5.70${}^{\#\#\#}$
**PCOS + Met**	58.40 ± 5.25*	64.60 ± 5.59${}^{\#\#\#}$	113.20 ± 10.09*	59.60 ± 5.17${}^{\#}$
**PCOS + NS 50**	35.80 ± 3.02${}^{\#\#\#}$	50.60 ± 4.01${}^{\#\#\#}$	87.80 ± 5.23${}^{\#}$	65.60 ± 4.52${}^{\#\#}$
**PCOS + NS 100**	53 ± 4.93#	68.60 ± 5.10${}^{\#\#}$*	94.40 ± 5.87	48.40 ± 6.40**
**PCOS + NS 200**	63.60 ± 5.22**	77.40 ± 5.58${}^{\#}$**	111 ± 8.21*	39.80 ± 3.38***
Data are indicated as Mean ± SEM. Statistical evaluation was done by analysis of variance (one-way ANOVA), followed by Tukey's test. A value of *p < 0.05, **p < 0.01, and ***p < 0.001 was considered as significant versus the PCOS control group; A value of ${}^{\#}$p < 0.05, ${}^{\#\#}$p < 0.01, and ${}^{\#\#\#}$p < 0.001 was considered as significant versus the intact control group, PCOS: Polycystic ovarian syndrome, Met: Metformin, NS: Nigella sativa seed, MDA: Malondialdehyde, SOD: Superoxide dismutase, GPX: Glutathione peroxidase, CAT: Catalase				

**Table 4:** Number of primordial, primary, secondary, graafian and atretic follicles, corpus luteum, and cyst in the ovarian tissues in the dehydroepiandrosterone-induced PCOS rats


[dir=NW,height=0.4in,width=0.9in,leftsep=0pt]**Groups** **Factors**	**Primordial follicles**	**Primary follicles**	**Secondary follicles**	**Graafian follicles**	**Corpus luteum**	**Atretic follicles**	**Cystic follicles**
**Control**	55.0 ± 1.51	28.0 ± 0.94	21.0 ± 0.82	7.2 ± 0.24	3.3 ± 0.12	5.7 ± 0.29	0.0 ± 0.0
**PCOS**	50.0 ± 1.47	24.0 ± 0.94	17.0 ± 0.61${}^{\#\#\#}$	5.0 ± 0.20${}^{\#\#\#}$	2.5 ± 0.12${}^{\#\#}$	7.0 ± 0.33	9.0 ± 0.24${}^{\#\#\#}$
**PCOS + Met**	50.0 ± 1.51	27.0 ± 0.94	19.0 ± 0.57	5.8 ± 0.57${}^{\#}$	2.7 ± 0.08${}^{\#}$	5.5 ± 0.24${}^{*}$	8.5 ± 0.37${}^{\#\#\#}$
**PCOS + NS 50**	51.0 ± 1.51	25.0 ± 0.98	17.0 ± 0.57${}^{\#\#\#}$	5.2 ± 0.57${}^{\#\#\#}$	3.6 ± 0.16${}^{***}$	5.5 ± 0.29${}^{*}$	8.5 ± 0.29${}^{\#\#\#}$
**PCOS + NS 100**	52.0 ± 1.39	23.0 ± 0.86${}^{\#\#}$	15.0 ± 0.53${}^{\#\#\#}$	5.1 ± 0.57${}^{\#\#\#}$	3.2 ± 0.12${}^{**}$	5.2 ± 0.33${}^{**}$	8.7 ± 0.29${}^{\#\#\#}$
**PCOS + NS 200**	53.1 ± 1.39	29.0 ± 0.94${}^{**}$	19.0 ± 0.53	7.0 ± 0.24${}^{***}$	3.0 ± 0.12	5.5 ± 0.37${}^{*}$	6.0 ± 0.29${}^{\#\#\#**}$
Data are indicated as Mean ± SEM. Statistical evaluation was done by analysis of variance (one-way ANOVA), followed by Tukey's test. A value of ${}^{*}$p < 0.05, ${}^{**}$p < 0.01, and ${}^{***}$p < 0.00 was considered as significant versus the PCOS control group; A value of ${}^{\#}$p < 0.05, ${}^{\#\#}$p < 0.01, and ${}^{\#\#\#}$p < 0.00 was considered significant versus the intact control group, PCOS: Polycystic ovarian syndrome, Met: Metformin, NS: *Nigella sativa *seed							

## 4. Discussion

In women with PCOS, the serum levels of testosterone, estradiol, and LH are increased and FSH and progesterone levels are decreased (17). In this study, the treatment of rats with DHEA elevated the LH, estrogen, and testosterone levels, and decreased the FSH and progesterone levels, which is consistent with the results of Walters study Walters study following the same model of inducing PCOS (20). Therefore, it seems that the factors which can lower LH and estrogen levels and increase the level of FSH and progesterone can be used to treat this disease.

The *N. sativa *seed, which is a plant about which the Prophet Muhammad (peace and blessings be upon him) said, “There is healing in the black seed for all diseases except death," was used in this study (21). In this study, the administration of *N. sativa *extract decreased the estrogen levels in the PCOS rats. Phytoestrogens are the active compounds of this extract which are able to, directly and indirectly, attach to estrogen receptors and cause significant estrogen-like effects (22). This effect, by negative feedback, reduces the plasma levels of estrogen. Also, the administration of the extract decreased the LH levels in PCOS rats. According to research, this may be related to linoleic acid (CLA) in *N. sativa*, which has a decreasing effect on LH levels. This effect of CLA is through the inhibition of nitric oxide and leptin, which are important controllers for the release of LH (23).

In DHEA-induced PCOS model, the co-occurrence of hyperandrogenism and decrease in FSH level, observed in our study, is very common. We believe that it can be claimed that the hydroalcoholic extract of *N. sativa *seed showed remarkable antiandrogenic effects by reducing elevated testosterone levels. This property of the extract is possibly due to the presence of phytoestrogens in the extract. More studies are required to elucidate the exact functional mechanism of the extract. Furthermore, increasing the level of androgens can increase the level of FSH receptors in PCOS patients (24), thus lowering the FSH serum level by negative feedback. Previous studies in PCOS indicate the relationship between the increased levels of LH or decreased FSH levels and creating insulin resistance.

Disruption in the usual hypothalamic-pituitary-gonadal axis increases both the levels of testosterone and LH leading to the disease state (24). LH stimulates testosterone secretion from ovarian theca cells through the PI3K/Akt pathway further elevating the activity of 17-a hydroxylase enzyme which catalyzes the conversion of progesterone to androgens, culminates in diminishing the progesterone level, and elevates the androgens level (25). Thus, the anti-androgenic effects of *N. sativa *seed may be due to its potentials obstructing the PK13 pathway. The results of the tests showed that the level of glucose, insulin, and insulin resistance significantly increased in the PCOS rats compared to the healthy control group. Reduction in serum glucose observed in the PCOS rats treated with *N. sativa* can occur for various reasons. Thymoquinone in *N. sativa *seed can reduce the expression of gluconeogenic enzymes and the production of hepatic glucose (26).

In addition, it has been shown that the liquid intake of *N. sativa *seed extract reduces glucose absorption and inhibits glucose carriers in diabetic rats (27). Our results verify the positive and dose-dependent effect of *N. sativa *seed in improving insulin resistance and serum level changes of insulin in PCOS rats. The findings suggests that hyperinsulinemia may enhance the chemerin gene expression in polycystic ovaries in which chemerin may play a role in pathophysiology of PCOS by direct action on ovary (28).

In addition, the mechanism responsible for insulin resistance is obscure. Insulin resistance is worsened by extreme fat mass or unusual secretion or function of adipocytokines such as chemerin (29). Furthermore, according to our results, in a human study it was shown that receiving *N. sativa* caused a decrease in the insulin resistance (26). In our study, the induction of PCOS led to a decrease in progesterone levels. This may be due to the expression of the chemerin gene which inhibited FSH-induced progesterone secretion in granulosa cells by prevention of aromatase and p450scc expression (30). The results of this study showed that as expected, metformin as standard drug for PCOS treatment could significantly decrease testosterone, LH, insulin resistance, and glucose serum level compared to the PCOS-induced groups. However, it seems that one of the mechanisms involved in the function of metformin in the treatment of PCOS is its effect on reducing the gene expression of chemerin, which has been reported in various studies (31). In our study, the weight of PCOS rats increased, and treatment with *N. sativa* caused a drop in weight. In a human study, similar to our research, two months of *N. sativa* administration to menopausal women led to weight loss compared to the PCOS group (32). study has indicated the anti-obesity results of *Nigella*, due to its affirmative effects against insulin sensitivity and its immune-modular effects (33).

In addition, *N. sativa* might possess anorectic effects and can cause a decrease in food intake. Despite these dates, more controlled intervention researches are necessary for higher comprehension the effects of *N. sativa* on weight loss. The increased androgens and insulin resistance can lead to oxidative stress (34), and the role of oxidative stress in PCOS pathogenesis can never be neglected (35). Therefore, reducing oxidative stress by increasing the activity of antioxidant enzymes can be one of the effective therapies in these patients. The results of this study showed that antioxidant enzymes levels increased and the malondialdehyde level decreased in rat's model of PCOS after treatment with the extract of *N. sativa* seed. This result is similar to the results of Leong and colleagues (36).

Recent studies have shown that thymoquinone in black seeds have inhibitory effects on free radicals (37, 38) and the potential antihyperglycemic properties of *N. Sativa *is based on its antioxidant content. Moreover, *N. sativa* is reported to stimulate paraoxonase enzyme, which functions as an antioxidant (27). Furthermore, flavonoids, active ingredients in *N. sativa* seed (21), have antioxidant properties (39). The results of hormonal analysis were also supported by histopathological findings of the uterus as histopathological changes reached up in the PCOS group relative to the control group. In PCOS rats follicular atresia increased.

Corpus luteum is a necessary factor for the synthesis of progesterone which affects the reproductive cycles and supports the uterus for implantation if conception happen, as in this study, in the PCOS group, the Corpus luteum was decreased accompanied by decline in progesterone level (40). Decrement in secondary follicles number because of the overproduction of androgens prevents normal follicular maturation process in PCOS group. However, the extract and metformin groups indicated a remarkable improvement of ovarian tissue with the appearance of reduction in cysts and outstanding regular luteinization (35).

## 5. Conclusion

The results of this study suggest that *N. sativa *seed extract has hypoglycemic, antioxidant effect and can modulate hormones related to fertility in PCOS rats. However, its use in the long-term treatment of PCOS, requires more investigation.

##  Conflict of Interest

The authors have no conflict of interest. The authors alone are responsible for the content and writing of the paper.
